# Smoking habits in French farmers: a cross-sectional study

**DOI:** 10.1186/s12889-017-4030-4

**Published:** 2017-02-04

**Authors:** Pauline Roux, Alicia Guillien, Thibaud Soumagne, Ophélie Ritter, Jean-Jacques Laplante, Cécile Travers, Jean-Charles Dalphin, Gérard Peiffer, Lucie Laurent, Bruno Degano

**Affiliations:** 1 Service d’Explorations Fonctionnelles Respiratoires, Centre Hospitalier Régional Universitaire (CHRU), Besançon, France; 2 Service de Pneumologie, CHRU, Besançon, France; 3 EA 3920, Université de Franche-Comté, Besançon, France; 4 EA4267, Université de Franche-Comté, Besançon, France; 5 Mutualité Sociale Agricole (MSA), Besançon, France; 6 Unité Mixte de Recherche, Centre National de la Recherche Scientifique Chrono-Environnement, Université de Franche-Comté, Besançon, France; 7 Service de Pneumologie-Tabacologie, CHR, Metz, France; 8 Physiologie-Explorations Fonctionnelles, CHU Jean Minjoz, Besançon Cedex, 25030 France

**Keywords:** Smoking, Farmers, Prevalence, Respiratory diseases

## Abstract

**Background:**

Farmers are exposed to multiple air contaminants that may interact with tobacco smoking in the development of respiratory diseases. Farmers are currently considered to smoke less than non-farmers, but precise data in different categories of age and farming activities are lacking.

**Methods:**

Smoking habits were studied in a cross-sectional study involving 4105 farmers and 996 non-farming controls aged 40–74 years in 9 French departments between October 2012 and May 2013. Three age groups were defined (40–54, 55–64 and 65-74years). Farmers were divided into four activity groups, namely cattle breeders, livestock farmers working in confined spaces, crop farmers and others. Smoking prevalence was compared between farmers and controls, and odds ratios (ORs) for smoking adjusted for age were calculated.

**Results:**

The adjusted OR for ever-smoking was lower among farmers than among non-farmers in all age categories, but the ORs for current smoking were similar in farmers and controls. Smoking prevalence varied according to the type of farming activity, and was lower than in non-farming controls only among cattle breeders and confined livestock farmers. In farmers, the proportion of smokers was higher in the youngest age categories compared with the older age classes.

**Conclusions:**

Our results confirm that the prevalence of ever-smokers is lower in farmers than in non-farmers. Nevertheless, our data show that active smoking prevalence is similar in farmers and in non-farmers. This suggests that farmers, just like non-farmers, should be targeted by primary prevention campaigns against smoking.

## Background

Farming activities count among the professions most at risk of acute and chronic respiratory diseases [1, 2]. The prevalence of chronic bronchitis, chronic obstructive pulmonary disease (COPD), hypersensitivity pneumonitis and toxic pulmonary diseases is significantly higher in farmers than in non-farmers [3]. This is likely related to the fact that farmers have lifelong exposure to multiple air contaminants (organic dusts, saprophytic microorganisms and/or chemical toxins) that may contribute to the development of respiratory diseases by allergic, inflammatory and/or pharmacological mechanisms [4, 5]. Tobacco smoking is in its own right a well-established risk factor for the development of chronic respiratory diseases, especially chronic bronchitis and COPD [6, 7]. Notably, there is an additive or even a synergistic effect on the decline in lung function and on the development of COPD [8] between tobacco and some occupational farming exposures. It therefore seems essential not only to prevent occupational exposure in farmers, but also to fight against tobacco smoking, especially in those who are exposed to noxious occupational airborne contaminants [9].

The prevalence of smoking conceals large disparities between professional sectors [10], and it has previously been reported that farmers smoke less than non-farmers [11, 12]. In a recent report by the Institut National de Prévention et d’Education pour la Santé (INPES), the prevalence of active smoking in France was estimated to be about 17% in farmers, while it was 23% in managers and 40% in manual workers [13]. In the French AGRICAN cohort (“AGRIculture and CANcer”) that included about 180,000 subjects working in the primary sector, the proportion of ever-smokers (either current or former smokers) was 58% in males and 24% in females [11]. In the general population, the INPES has reported a higher proportion of ever-smokers than that observed in farmers included in the AGRICAN cohort, with a prevalence of 64% in males and 51% in females [14].

Active tobacco smoking is increasing again after 20 years of decline. For example, the prevalence of active smoking in the French working population increased from 30.8% in 2005 to 33.2% in 2010 [13]. The same also appears to be true in farmers, with smoking prevalence increasing from 12.5% in 2005 to 19.0% in 2010 [13]. Although the prevalence of COPD varies largely from one farming activity to another [2], the prevalence of smoking has not recently been evaluated in detail in agricultural workers. The aim of the present work was therefore to study the smoking prevalence in different agricultural sectors and to compare with non-farmers.

## Methods

### Study population

**Fig. 1:**
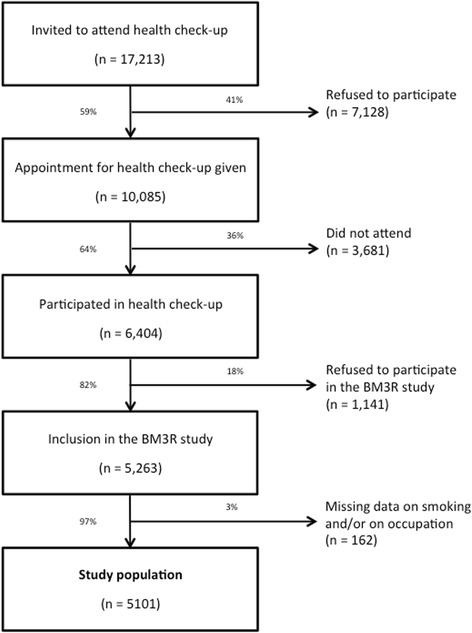
Flow chart of participants included in the study

Data for this study were collected as part of the BM3R project (BM3R: BPCO MSA 3 Régions) that was conducted between October 2012 and May 2013 in 3 French regions comprising 9 French departments (Doubs, Haute-Saône, Jura, Territoire de Belfort, Finistère, Ille-et-Vilaine, Côtes-d’Armor, Morbihan and Gironde) in collaboration with the MSA (Mutualité Sociale Agricole), which is the French national social security system to which all agricultural workers are affiliated. These three regions (Bretagne, Aquitaine and Franche-Comté) are located in different geographical areas of France (northwest, southwest and east, respectively). These regions were opportunistically selected, without any hypothesis regarding tobacco smoking prevalence. Data were collected among affiliated members (farmers and administrative workers of both genders) who agreed to participate. During the study period, 17,213 subjects aged 40–75 years were invited to attend a free health check-up performed in a premises close to their home. In total, 37% of the invited MSA members participated in the check-up. Among those who participated in the check-up, 82% accepted to participate in the BM3R project (Fig. 1). During the check-up, each participant completed a questionnaire (in French) that included questions regarding smoking and professional histories.

Ethical approval was received from the local Ethics Committee (CPP Est; under the number 13–682), and written consent was obtained from all participants.

### Questionnaire

Questions regarding the participant’s smoking history included the number of cigarettes/pipe/cigars smoked per day and the dates when smoking was started and given up. From these data, the number of pack-years was calculated and smoking status was defined: non-smokers were defined as those having smoked on average less than one cigarette, one cigar or one pipe a day for a year. Current smokers smoked this amount or more, and ex-smokers were defined as those who had stopped smoking at least 1 month before the time at which they completed the questionnaire [15]. The group of ever-smokers comprised ex-smokers plus current smokers.

Data regarding professional history included the last five jobs held by the participants, with the start and finish dates for each job. Occupational categories include employees, self-employed and retired persons. Participants who declared having worked only in non-agricultural jobs without any known exposures were used as controls (tertiary sector). Among farmers (primary sector), three specific groups were defined: exclusive crop farmers (growing cereals, fruit, potatoes,…); cattle breeders (either dairy farmers or meat producers); and livestock farmers working in confined spaces (swine breeders; poultry breeders and farmers breeding two or more types of livestock). To be classified in one of these three groups, participants must have worked at the specified job for at least 10 years. To be classified in the breeders group, participants must have raised only the specified livestock, but could also work or have worked as crop farmers and/or worked in a job without any known exposure. All participants who did not meet the criteria for classification in one of the three specific groups defined above were classified as “other” farmers, as previously described [2].

### Statistical analysis

Comparisons of smoking prevalence between professional groups and subgroups were performed using the Chi-squared test for qualitative data and ANOVA for quantitative data. When a difference was found, we used a Student’s t-test with Bonferroni correction. The variables age and tobacco habits were divided into three categories (<55 years, 55–64 years, ≥65 years; and never smokers, former smokers, current smokers, respectively). Odds ratios (ORs) were computed by logistic regression and adjusted for gender, since gender has been demonstrated to be strongly associated with tobacco habits [16, 17]. A *p*-value less than 0.05 was considered statistically significant. All data were analysed using SAS software (version 9.3; SAS Institute, Inc., Cary, NC, USA).

## Results

### Population characteristics

A total of 5263 subjects were included in the study. Among these, 162 were excluded from further analysis because of missing data either regarding smoking habits and/or occupation. The main characteristics of the 5101 remaining participants are shown in Table 1. Primary sector professions comprised 595 (14.5%) crop farmers, 1687 (41.1%) cattle breeders, 772 (18.8%) livestock farmers and 1051 (25.6%) others. The tertiary sector comprised 996 non-farming participants. The proportion of men was higher among farmers (62.5%) than among non-farmers (45.3%). Although mean age was similar in farmers and non-farmers (Table 1), men were slightly, yet statistically significantly younger than women (56.4 ± 8.6 vs. 57.0 ± 9.0 years, respectively; *p* = 0.03).

**Table 1:** Characteristics of the study population and distribution according to sector of activity

	Total	Activity sector		
	Population	Tertiary sector	Primary sector	
	(*n* = 5101)	(*n* = 996)	(*n* = 4105)	*p*
Men, n (%)	2995 (59%)	449 (45%)	2546 (62%)	<0.0001
Mean age (years)	56.9 ± 9.0	56.4 ± 8.6	57.0 ± 9.0	0.07
Age category, *n* (%)				0.12
40–54 years	1946 (38%)	386 (39%)	1560 (38%)	
55–64 years	1677 (33%)	349 (35%)	1328 (32%)	
65–74 years	1478 (29%)	261 (26%)	1217 (30%)	
Body Mass Index (kg/m^2^)	26.6 ± 4.6	25.7 ± 4.4	26.9 ± 4.6	<0.0001
Smoking status, *n* (%)				<0.0001
Never smoker	3324 (65%)	578 (58%)	2746 (67%)	
Former smoker	1082 (21%)	269 (27%)	813 (20%)	
Current smoker	695 (14%)	149 (15%)	546 (13%)	
Mean pack-years	6.2 ± 13.2	7.2 ± 14.3	6.0 ± 12.9	0.02

Data are presented as *n* (%) or mean ± SD, unless otherwise stated

### Smoking prevalence

The prevalence of ever- and current smokers according to age in farmers (primary sector) and in controls without any occupational exposure (tertiary sector) is summarized in Table 2. Among the controls from the tertiary sector, the prevalence of ever-smokers was similar in all age categories. In contrast, the prevalence of ever-smokers was significantly higher in the two youngest age categories than in the oldest one in farmers (Table 2). After adjustment for gender, the odds ratios for being an ever-smoker (i.e., either current or former smoker) were lower in farmers than in controls from the tertiary sector in all age categories.

**Table 2:** Prevalence of smokers (ever and current) according to age intervals and activity sectors

	Ever smokers		Current smokers	
	Prevalence	OR [95% CI]	Prevalence	OR [95% CI]
40–54 years				
Tertiary sector	186 (48%)	1.00	82 (21%)	1.00
Primary sector	670 (43%)	**0.73 [0.58–0.91]**	337 (22%)	0.92 [0.70–1.22]
55–64 years				
Tertiary sector	146 (42%)	1.00	49 (14%)	1.00
Primary sector	441 (33%)^*^	**0.57 [0.44–0.74]**	160 (12%)	0.76 [0.54–1.08]
65–74 years				
Tertiary sector	86 (33%)	1.00	18 (7%)	1.00
Primary sector	248 (21%)^*^	**0.39 [0.28–0.54]**	49 (4%)^*^	0.98 [0.74–1.30]

Data are presented as *n* (%) or OR (95% CI). Data presented in bold are statistically significant. ^*^ *p* < 0.05 versus tertiary sector for prevalence. Odd ratios (*ORs*) are adjusted for gender

The adjusted odds ratio for being a current smoker was similar in farmers and in controls from the tertiary sector (Table 2). In addition, the prevalence of current smokers was significantly higher in the youngest age category as compared to the oldest age group in farmers.

The analysis of smoking prevalence in the 4 pre-specified subgroups of farming professions is shown in Table 3. In the oldest age category (those aged 65–74 years), the adjusted odds ratios were lower in the 4 pre-specified subgroups of farmers as compared with controls from the tertiary sector. By contrast, in the youngest age categories (40–54 and 55–64 years), there was lower prevalence and lower odds ratios for smoking only among cattle breeders and livestock farmers working in confined spaces as compared with controls.

**Table 3:** Prevalence of smokers (ever and current) according to age category and type of farming activity

	Ever smokers		Current smokers	
	Prevalence	OR [95% CI]	Prevalence	OR [95% CI]
40–54 years				
Tertiary sector	186 (48%)	1.00	82 (21%)	1.00
Cattle	192 (33%)	**0.48 [0.36–0.63]**	90 (16%)	**0.60 [0.42–0.85]**
Confined	111 (41%)	**0.66 [0.48–0.91]**	47 (17%)	0.73 [0.48–1.09]
Crop	138 (60%)	0.97 [0.71–1.33]	82 (30%)	1.48 [1.03–2.13]
Others	229 (53%)	1.12 [0.84–1.48]	118 (28%)	1.34 [0.96–1.87]
55–64 years				
Tertiary sector	146 (42%)	1.00	49 (14%)	1.00
Cattle	133 (26%)	**0.40 [0.29–0.54]**	48 (9%)	**0.59 [0.38–0.92]**
Confined	86 (31%)	**0.51 [0.36–0.73]**	27 (10%)	0.63 [0.38–1.06]
Crop	90 (44%)	0.95 [0.66–1.38]	33 (16%)	1.04 [0.62–1.72]
Others	132 (40%)	0.74 [0.53–1.02]	52 (16%)	1.07 [0.69–1.66]
65–74 years				
Tertiary sector	86 (33%)	1.00	18 (7%)	1.00
Cattle	93 (16%)	**0.31 [0.21–0.46]**	19 (3%)	**0.41 [0.21–0.82]**
Confined	33 (15%)	**0.28 [0.17–0.47]**	5 (2%)	**0.25 [0.08–0.74]**
Crop	30 (26%)	**0.46 [0.27–0.80]**	3 (3%)	**0.28 [0.08–0.96]**
Others	92 (31%)	0.70 [0.47–1.03]	22 (8%)	0.89 [0.46–1.73]

Data are presented as *n* (%) or OR (95% CI). Data presented in bold are statistically significant. Odd ratios (*ORs*) are adjusted for gender

## Discussion

The main findings of this study are that: (1) smoking prevalence was lower among farmers than among non-farmers; (2) this prevalence depended on the farming activity, and was lower than in non-farmers only among cattle breeders and livestock farmers working in confined spaces; (3) among farmers, the proportion of smokers was higher in the youngest age categories compared with the oldest age group.

Our study of a large sample of French farmers suggests that farmers are more likely to have never smoked than non-farmers. The prevalence of never-smokers observed here in farmers is similar to that reported in a study performed among 755 Polish farmers whose age was similar to that of our participants [18]. In the recently published “Irish farmers lung health study” [19], all study participants were farming volunteers attending an agricultural exhibition. Data from 372 farmers were analysed. The majority of participants were male (76%) and 61% were never smokers. In a study performed in a rural region of upstate New York comparing farmers and rural non-farming residents, farmers had lower rates of smoking (OR: 0.60, 95% CI: 0.40–0.89) than non-farmers after adjustment for age, gender, education and having a regular health care provider [20]. In the French AIRBAg study [21] that enrolled 277 dairy farmers (69% men), the proportion of never smokers was 71%, and ever-smokers had predominantly moderate tobacco consumption (<10 pack-years). However, in our study, we observed a lower prevalence of smokers among farmers than previously reported by others. For example, the prevalence of active smokers reported by the INPES in 2010 in subjects aged 15–85 years was 32% in the general population, and 19% in farmers [14]. The fact that our study did not include subjects aged 15–39 years, an age category in which active smoking prevalence is high [22], likely explains the higher proportion of smokers in the INPES series compared to our study.

We also confirm findings reported by others indicating that the prevalence of current smoking is higher in the younger age categories compared to the older age groups [23]. In our study, this observation was particularly true in farmers. Ever-smokers are more vulnerable to disease and death throughout life. There could therefore exist a selection bias with increasing age, with the result that the proportion of healthy never-smoking subjects was higher among older participants. Nevertheless, such a bias would apply for both farmers and non-farming controls. The youngest farmers are also those who are likely to be exposed for decades to occupational airborne pollutants. As it is likely that the combination of tobacco smoking and occupational exposures has an additive or even synergistic effect on COPD [8], it seems important to initiate smoking prevention strategies in active farmers.

We believe that an original finding of our study is that smoking prevalence varies significantly from one farming subgroup to another. This has previously been observed in the French AGRICAN cohort, in which smoking was more frequent among agricultural workers than among farm managers [24]. In our study, the two subcategories with the lowest smoking prevalence were those with the highest prevalence of COPD [2]. Although our study was not designed to address this point, it is plausible that farmers whose occupation is associated with a higher risk of respiratory diseases never smoke, or quit smoking at an early age. Nevertheless, we also report a high prevalence of COPD in the subgroup of “other” farmers, which is very heterogeneous in terms of farming activities, and thus in terms of occupational exposures [2]. This subgroup had a smoking prevalence that was similar to the smoking prevalence of controls from the tertiary sector.

### Limitations of the study

Although our study included a large number of controls from the tertiary sector as well as large population of farmers from many farming sectors, we acknowledge that the estimation of smoking prevalence could have been biased by the fact that only a fraction of the MSA members invited to the health check-up actually participated in the survey. According to the design of the study, the characteristics of the subjects who did not participate are not known. The participation rate in a previous round of health check-ups organized by the MSA in 2011, as well as the characteristics of participants and non-participants, has recently been studied. In an analysis of 27,848 invited subjects (mean age 60.7 years), the participation rate was 39.4% [25], a value that is very close to the participation rate in the present study. A hierarchical cluster analysis of all invited subjects identified 2 groups of non-participants (namely, men in good health who are low users of health care [42% of all invited subjects]; and secondly, men and women in poor health who are high users of health care [22% of all invited subjects]), as well as 2 main groups of participants (namely, men in good health who are low users of health care except for health check-ups [16% of all invited subjects]; and secondly, women in good health who are high users of health care [20% of all invited subjects]). Tobacco habits of non-participants were unfortunately not investigated.

Another limitation is the lack of information on educational level and income levels. Indeed, it has been demonstrated in populations other than farmers that annual household income and educational level are negatively associated with the probability of nicotine dependence [26]. We also acknowledge that smoking prevalence might have been underestimated by self-reported smoking status, and that this potential misclassification could have been overcome by cotinine measures in biological fluids [27].

## Conclusions

This analysis of recent data collected in a large population comprising farmers and controls from the tertiary sector (non-farming controls) indicates that the prevalence of smoking is lower in farmers than in non-farmers. Nevertheless, the prevalence of smoking is not uniform across all farming categories, and is similar in crop farmers and in non-farming controls. In addition, our analysis suggests that smoking prevalence may be on the increase in farmers. Taken together, these results suggest that farmers should be targeted for primary prevention campaigns against smoking, especially as this population has an elevated risk of respiratory diseases.

## Abbreviations

COPD: Chronic obstructive pulmonary disease
CPP: Comité de Protection des Personnes
INPES: Institut National de Prévention et d’Éducation pour la Santé
MSA: Mutualité Sociale Agricole
ORs: Odds ratios
